# Understanding the importance of low‐molecular weight (ethylene oxide‐ and propylene oxide‐induced) DNA adducts and mutations in risk assessment: Insights from 15 years of research and collaborative discussions

**DOI:** 10.1002/em.22248

**Published:** 2018-12-10

**Authors:** L. H. Pottenger, G. Boysen, K. Brown, J. Cadet, R. P. Fuchs, G. E. Johnson, J. A. Swenberg

**Affiliations:** ^1^ Olin Corporation/Blue Cube Operations, LLC, retired, LHP TOX CONSULT, LLC Midland MI USA; ^2^ Department of Environmental and Occupational Health and The Winthrop P Rockefeller Cancer Institute University of Arkansas for Medical Sciences Little Rock Arkansas USA; ^3^ Leicester Cancer Research Centre University of Leicester Leicester United Kingdom; ^4^ Institut Nanosciences et Cryogénie, CEA‐Grenoble Grenoble France; ^5^ Université de Sherbrooke Sherbrooke Canada; ^6^ Centre de Recherche en Cancérologie de Marseille (CRCM), Inserm, U1068 Marseille, 13009 France; ^7^ CNRS, UMR7258 Marseille, 13009 France; ^8^ Institut Paoli‐Calmettes Marseille, 13009 France; ^9^ Aix‐Marseille University UM 105, 13284, Marseille France; ^10^ Swansea University, Institute of Life Sciences Swansea United Kingdom; ^11^ University of North Carolina Chapel Hill North Carolina USA

**Keywords:** DNA adducts, genotoxic effects, dose‐response, mutations, N7‐alkyl/hydroxyalkylguanine adducts

## Abstract

The interpretation and significance of DNA adduct data, their causal relationship to mutations, and their role in risk assessment have been debated for many years. An extended effort to identify key questions and collect relevant data to address them was focused on the ubiquitous low MW *N*7‐alkyl/hydroxyalkylguanine adducts. Several academic, governmental, and industrial laboratories collaborated to gather new data aimed at better understanding the role and potential impact of these adducts in quantifiable genotoxic events (gene mutations/micronucleus). This review summarizes and evaluates the status of dose–response data for DNA adducts and mutations from recent experimental work with standard mutagenic agents and ethylene oxide and propylene oxide, and the importance for risk assessment. This body of evidence demonstrates that small *N*7‐alkyl/hydroxyalkylguanine adducts are not pro‐mutagenic and, therefore, adduct formation alone is not adequate evidence to support a mutagenic mode of action. Quantitative methods for dose–response analysis and derivation of thresholds, benchmark dose (BMD), or other points‐of‐departure (POD) for genotoxic events are now available. Integration of such analyses of genetox data is necessary to properly assess any role for DNA adducts in risk assessment. Regulatory acceptance and application of these insights remain key challenges that only the regulatory community can address by applying the many learnings from recent research. The necessary tools, such as BMDs and PODs, and the example datasets, are now available and sufficiently mature for use by the regulatory community. Environ. Mol. Mutagen. 60: 100–121, 2019. © 2018 The Authors. *Environmental and Molecular Mutagenesis* published by Wiley Periodicals, Inc. on behalf of Environmental Mutagen Society.

## INTRODUCTION

The appropriate interpretation and application of DNA adduct data to inform risk assessment decisions have been debated for the past several decades. The intensity of this debate has increased more recently with the advent of new, highly specific, and exquisitely sensitive, analytical techniques available to quantify DNA adduct data and the remarkable insights stemming from new data obtained with those techniques (Gocke and Müller, 2009, Nakamura et al., 2014, Swenberg et al., 2011). The growing wealth of structurally quantitative data on DNA adducts—both those exogenously induced and those endogenously present from normal metabolic processes, including background exposures from diet, *etc*.—provides impetus for an examination of the role of DNA adducts in the determination of modes of action (MOA) for adverse outcomes. Indeed, evaluation of appropriate dose–response models for the key events in question is also critical. The clear impact of MOA on regulatory decisions *vis‐à‐vis* linear *vs*. non‐linear dose–response modelling, in particular, emphasizes the importance of this issue. Current US EPA regulatory guidance imposes a linear dose–response model to determine risk below the identified point of departure (POD; see text box) when a mutagenic MOA (sometimes described as a DNA‐reactive MOA) is established for a chemical (U.S. Environmental Protection Agency, 2005). In fact, current practice often, and quite erroneously, ascribes a mutagenic MOA to any chemical that causes DNA adducts (often only inferred from a positive *in vitro* genetox battery), without the rigorous evaluation of levels of evidence that such a determination requires. A thorough approach was described by an International Life Sciences Institute/Health and Environmental Sciences Institute (ILSI/HESI) Committee (Jarabek et al., 2009), which emphasized the role of DNA adducts as biomarkers of exposure. This and other approaches have been applied to some chemicals, offering case studies (Manjanatha et al., 2017, Pottenger et al., 2014). Indeed, determination of a mutagenic MOA for cancer cannot depend solely on a positive *in vitro* genetox battery but requires a demonstrated causal link between adduct formation and induction of *in vivo* mutations in target tissue in cancer‐related genes (Moore et al., 2018). Thus, an assessment of a chemical that forms DNA adducts (either specifically quantified or inferred based solely on positive *in vitro* genetox data), can incorrectly conclude it has a mutagenic MOA, leading to an inappropriate linear extrapolation of dose–response below the POD, which may result in overestimating the actual risk at low dose.

**Terminology Related to Dose–Response Modeling**

**BenchMark Dose** (BMD): modelled dose levels corresponding to specific response levels, or benchmark responses, near the low end of the observable range of the data, which serve as possible points of departure (PODs) for linear or nonlinear extrapolation of health effects data and/or as bases for comparison of dose–response results across studies/chemicals/endpoints (U.S. Environmental Protection Agency, 2012). The BMD is estimated by fitting a series of dose–response models to data, and selecting the best fitting model. An approximate lower confidence bound of the BMD (BMDL) is suggested to replace the NOAEL as a point of departure in the health risk assessment of chemical substances.

**Break Point Dose** (BPD): Similar to Td, the dose–response model determines the best‐fitting two‐segment linear function where the first segment from zero dose to the breakpoint is horizontal (i.e., has zero slope) and the second segment has a positive slope. The inflection point where the two lines meet is termed the BPD (Johnson et al., 2014).

**No Observed Genotoxicity Exposure Level** (NOGEL): Analogous to a no‐adverse‐effect‐level (NOAEL) value, the NOGEL value is defined as the highest dose in a genotoxicity assay (*in vitro* or *in vivo*) that does not differ statistically from the control value.

**Point of Departure** (POD): It is defined as the point on a toxicological dose–response curve established from experimental data or observational data generally corresponding to an estimated low observed effect level (LOEL) or no observed effect level (NOEL). A POD marks the beginning of extrapolation to a toxicological reference dose (RfD) or reference concentration (RfC). The POD is an estimated dose near the lower end of the observed range, without significant extrapolation to lower doses (U.S. Environmental Protection Agency, 2005). Extrapolation below the POD can define an eventual risk value; safety factors may (or may not) be applied to a POD.

**Threshold:** The use of the term “threshold” to describe a dose–response curve in this paper is considered to be interchangeable with the term bilinear; it indicates that there are responses in treated systems (cells, tissues, *etc*.) that are not quantifiably different from control/background responses and responses that are quantifiably different from control/background responses. Such quantifiable differences generally are statistically identified as significantly different from those that are not quantifiably different from control/background values, although not always. Thus, at doses below a “threshold” no (statistically) quantifiable increase is measured; at doses above a “threshold”, there is a (statistically) quantifiable difference.

**Threshold dose** (Td): A Td value is statistically defined by applying a bilinear dose–response model to a dataset, for which the lower limit of the confidence interval must be positive (nonzero) (Lutz and Lutz, 2009). The statistical package compares fit for the bilinear (Td) model to a linear model and determines which one is a better statistical fit for the data being modelled, and when appropriate, the Td value.

Indeed, the available information has grown substantially in the past 15+ years and has revealed the ubiquitous presence of endogenous/background DNA adducts, including pro‐mutagenic adducts. In conjunction with new data on DNA adduct chemistry and fate, including DNA repair, these provide important new perspectives for understanding the biological consequences and biological significance of specific adducts and how to incorporate this information into risk assessment. This review focuses on research conducted from 2001–2016 and aims to provide a better understanding of specific questions pertaining to *N*7‐guanine adducts formed from exposure to low molecular weight (LMW) chemicals, including Ethylene (CAS 74‐85‐1)/Ethylene Oxide (CAS 75‐21‐8) (E/EO) and Propylene (CAS 115‐07‐1)/Propylene Oxide (CAS 75‐56‐9) (P/PO). Much of the experimental work was conducted on recognized, direct‐acting mutagens: ethyl methane sulfonate (EMS), methyl methane sulfonate (MMS), ethyl nitrosourea (ENU), and methyl nitrosourea (MNU), all also LMW chemicals and typically employed as positive controls. This article provides some background on LMW adducts and mutations, followed by an overview of our now completed research projects, a summary of what was learned and how it complements other research conducted with similar focus on the dose–response for mutation induction and the question of thresholds in mutation response to DNA adducts, and a description of several international workshops and committees’ efforts to integrate these learnings, ending with a look at a path forward. All of this provides context to the current understanding of the biological significance of *N*7‐alkyl/hydroxyalkylguanine (*N*7‐alkylG) adducts for risk assessment.

## OVERVIEW OF ADDUCT FORMATION, STABILITY, AND FATE: LMW DNA ADDUCTS

### Chemistry, Stability, and Adduct Dose‐Response of LMW DNA Adducts

**Figure 1:**
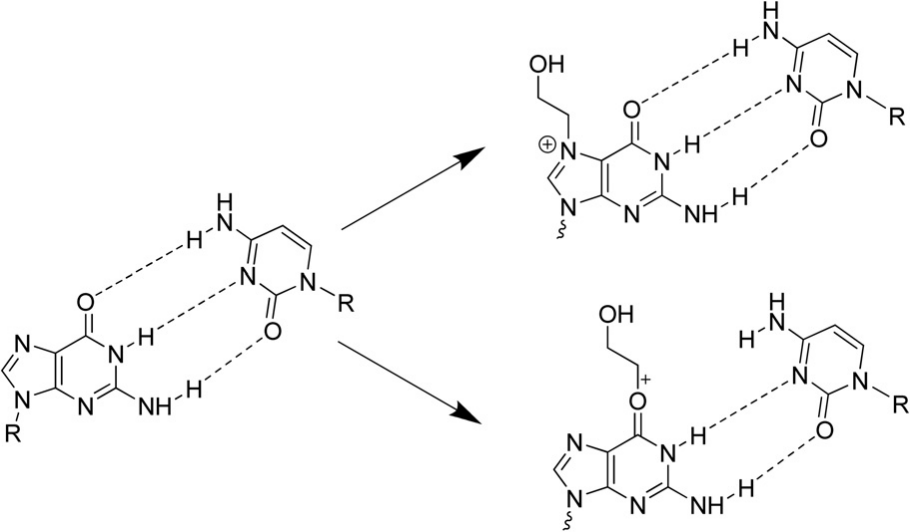
Structures of guanine‐cytosine base pairs and ethylene oxide (EO)‐induced adducts: *N*7‐HEG and O^6^‐HEG (S_N_2 reaction). Disrupted hydrogen bonding (− − ‐) shown for O^6^‐HEG, which is unaffected for *N*7‐HEG. *N*7‐HEG adducts comprise the majority (>95%) of EO‐induced adducts.

Beginning in the late 1940s/early 1950s, Miller and Miller pioneered the field of chemical carcinogenesis and were the first to demonstrate covalent binding of chemical carcinogens to macromolecules by reaction with nucleophilic sites (electron‐rich moieties, S, N, and O) in DNA and proteins (Miller and Miller, 1947, Wheeler and Skipper, 1957). Subsequent *in vitro* and *in vivo* studies soon demonstrated that, under physiological conditions (pH 7.4, 37°C), alkylation of DNA primarily occurred at the *N*7‐position of guanine (Brookes and Lawley, 1961) (Figure 1). Covalent binding to DNA was shown to occur mainly *via* either monomolecular (S_*N*_1, *e.g*., nitrogen mustards) or bimolecular nucleophilic (S_*N*_2, *e.g*., sulfonyl esters) substitutions (Brookes and Lawley, 1961, O'Connor, 1981, Swenson, 1983, Swenson et al., 1986). Reactive agents acting *via* S_N_1 reactions attack more frequently at the O^6^ position of guanine (although still a minor proportion of total adducts), which disrupts hydrogen bonding, thus producing a higher proportion of the pro‐mutagenic O^6^‐guanine adducts, compared to agents that react solely *via* the S_N_2 mechanism (Beranek, 1990, Singer, 1972, Singer et al., 1981) (Fig. 1).

Compared to many other DNA adducts, *N*7‐guanine adducts are chemically unstable, with half‐lives in double‐stranded DNA (dsDNA) ranging from as little as 2 h to 150 h (Margison et al., 1976). For example, the half‐life values for the *N*7‐(2‐hydroxypropyl)guanine (*N*7‐HPG) adduct in rat nasal respiratory epithelium, lung, and liver tissues are 5.5, 5.8, and 6.5 hrs, respectively (Osterman‐Golkar et al., 2003). The particular instability of *N*7‐guanine adducts is due to the additional positive charge placed on the guanine ring system. In general, larger alkyl groups promote spontaneous depurination in dsDNA (King et al., 1979, Margison et al., 1976, Osborne and Merrifield, 1985). Indeed, unlike most other DNA adducts, many *N*7‐guanine adducts undergo spontaneous depurination. In addition, they can accumulate in DNA with continuous exposure or treatment, but usually reach a plateau (steady state) after ~7–10 days of repeated exposure (Doerge et al., 2005, Lewis and Swenberg, 1980, Walker et al., 2000, Young et al., 2007). For example, steady state for the *N*7‐(2‐hydroxyethyl)guanine (*N*7‐HEG) adduct is reached following 7–10 days of repeated exposure to EO (Walker et al., 2000). At steady state, the number of *N*7‐HEG adducts formed is equal to the number of adducts lost due to depurination, repair, or cell death. In contrast, adducts that are more persistent, such as O^4^‐ethylthymine (O^4^‐ET), continued to accumulate over an exposure period of 4 weeks (Boucheron et al., 1987), and O^6^‐methylguanine (O^6^‐MG) in the brain (where repair is lacking) continued to accumulate over 6 weeks of dosing (Kleihues and Bucheler, 1977). The addition of a positive charge on the guanine ring system also promotes further reactions that have been reviewed in detail by Gates et al. (2004). The main reactions characteristic for *N*7‐guanine adducts include: (i) loss of C‐8 proton, (ii) (spontaneous) depurination, (iii) imidazole ring opening to yield N^5^‐alkyl‐2,6,‐diamino‐4‐hydroxy‐5‐formamidopyrimidine (alkyl‐FAPy), (iv) hydrolysis of the *N*7‐alkyl bond, and (v) rearrangement to C8 adducts.

Relative to guanine itself, *N*7‐methylguanine (*N*7‐MG) depurinates 10^6^ times more rapidly at pH 7, 37°C (Gates et al., 2004). The frequent spontaneous depurination of *N*7‐guanine adducts leaves behind an abasic or an apurinic/apyrimidic site (AP site), which is a noninformational lesion that is recognized as pro‐mutagenic (Schaaper and Loeb, 1981, Neto et al., 1992). In yeast cells, C is preferentially inserted across endogenously generated AP sites, followed by the insertion of A, and less frequently of T (Auerbach et al., 2005). Thus, for an *N*7‐MG‐induced AP site, C insertion is nonmutagenic, while A insertion leads to G:C to T:A (G➔ T) transversions. Although this process has been proposed as a possible pathway to induce a mutation from an *N*7‐guanine adduct, at least two attempts to provide evidential support were not successful, with no clear increases in AP sites identified in DNA heavily adducted with *N*7‐guanine adducts (Rios‐Blanco et al., 2000, Rusyn et al., 2005).

Opening of the imidazole ring of *N*7‐alkyl/hydroxyalkylguanine adducts gives rise to related N^5^‐alkyl/hydroxyalkyl‐2,6‐diamino‐4‐hydroxy‐5‐formamidopyrimidine adducts (N^5^‐alkyl/hydroxyalkyl‐FAPy) (Pujari and Tretyakova, 2017). This reaction is promoted under alkaline conditions. Many of these FAPy adducts have been chemically characterized with model compounds (Pujari and Tretyakova, 2017). However, the formation of most N^5^‐substituted FAPy derivatives remains to be established in cellular DNA. While data for some large, bulky FAPy adducts, for example, aflatoxin B_1_, indicate the FAPy adduct derived from *N*7‐AFB_1_‐G is more stable than the originating adduct (Brown et al., 2006, Moore et al., 2018), similar data were not identified on the frequency or stability of potential FAPy adducts from the smaller, LMW adducts addressed in this review.

There is a wealth of data on the formation and dose–response of LMW *N7*‐alkylG adducts in animal models. Metabolic activation of E or P to their respective oxides, EO or PO, *via* CYP450 activity occurs in all mammalian tissues investigated. The ubiquitous nature of these oxides, especially EO, is due to the ubiquitous nature of the parent olefin, E (and to a lesser degree P), present in natural/endogenous systems as a product of intermediary metabolism and from background natural and anthropogenic sources (Swenberg et al., 2008).

The reactive epoxide metabolites can bind to DNA and form *N*7‐HEG (Fig. 1) or *N*7‐HPG as the major DNA adduct (>95%). Indeed, there is an endogenous/background level of these same *N*7‐guanine adducts in all animal and human tissues investigated (Swenberg et al., 2011), formed by ubiquitous exposure to endogenous and other sources of the parent olefin/oxide. These sources include food, gut bacteria, and normal metabolic processes, in addition to combustion (*e.g*., forest fires, volcanos) and anthropogenic sources. The ubiquitous presence of endogenous/background *N*7‐guanine adducts must be considered and addressed in any MOA‐based risk assessment and evaluation of low exposures to DNA‐reactive chemicals, especially in decisions on selection of appropriate dose–response models and PODs (Swenberg et al., 2008).

The fate of these *N*7‐guanine adducts influences the adduct dose–response curves obtained using the very sensitive techniques available. Inhalation studies with E or P in mice and rats have established adduct dose–response curves supportive of the saturation of CYP450‐mediated metabolic activation to their respective reactive oxides, which then form the DNA adducts (Bolt et al., 1984, Bolt and Filser, 1987, Wu et al., 1999, Pottenger et al., 2007, Rusyn et al., 2005). For example, the metabolism of E to EO in mice saturates at ~1000 ppm E (Bolt et al., 1984, Bolt and Filser, 1987, Wu et al., 1999, Rusyn et al., 2005), producing in liver ~3.5 *N*7‐HEG/ 10^7^ normal nucleotides (Filser et al., 1992, Walker et al., 1993). In contrast, direct exposure to the oxide metabolites produces a linear dose–response for *N*7‐HEG/*N*7‐HPG (Filser and Bolt, 1983, Filser et al., 1992, Rios‐Blanco et al., 2000, Walker et al., 1993, 2000). The molecular dose of *N*7‐HEG can be orders of magnitude greater for exposures to EO than can ever be achieved by E exposure alone. In subacute or chronic exposures to EO, *N*7‐HEG adducts increase daily until they reach a steady state after 7–10 days (Walker et al., 1993, 2000). This nonlinear response over time is attributed to the chemical instability of *N*7‐HEG and on‐going spontaneous depurination. Similar kinetics have been demonstrated for P *vs*. PO exposures (Filser et al., 2000, 2001, Rios‐Blanco et al., 2000, 2003, Csanády and Filser, 2007, Pottenger et al., 2007).

### Mutation Induction by LMW Adducts

**Figure 2:**
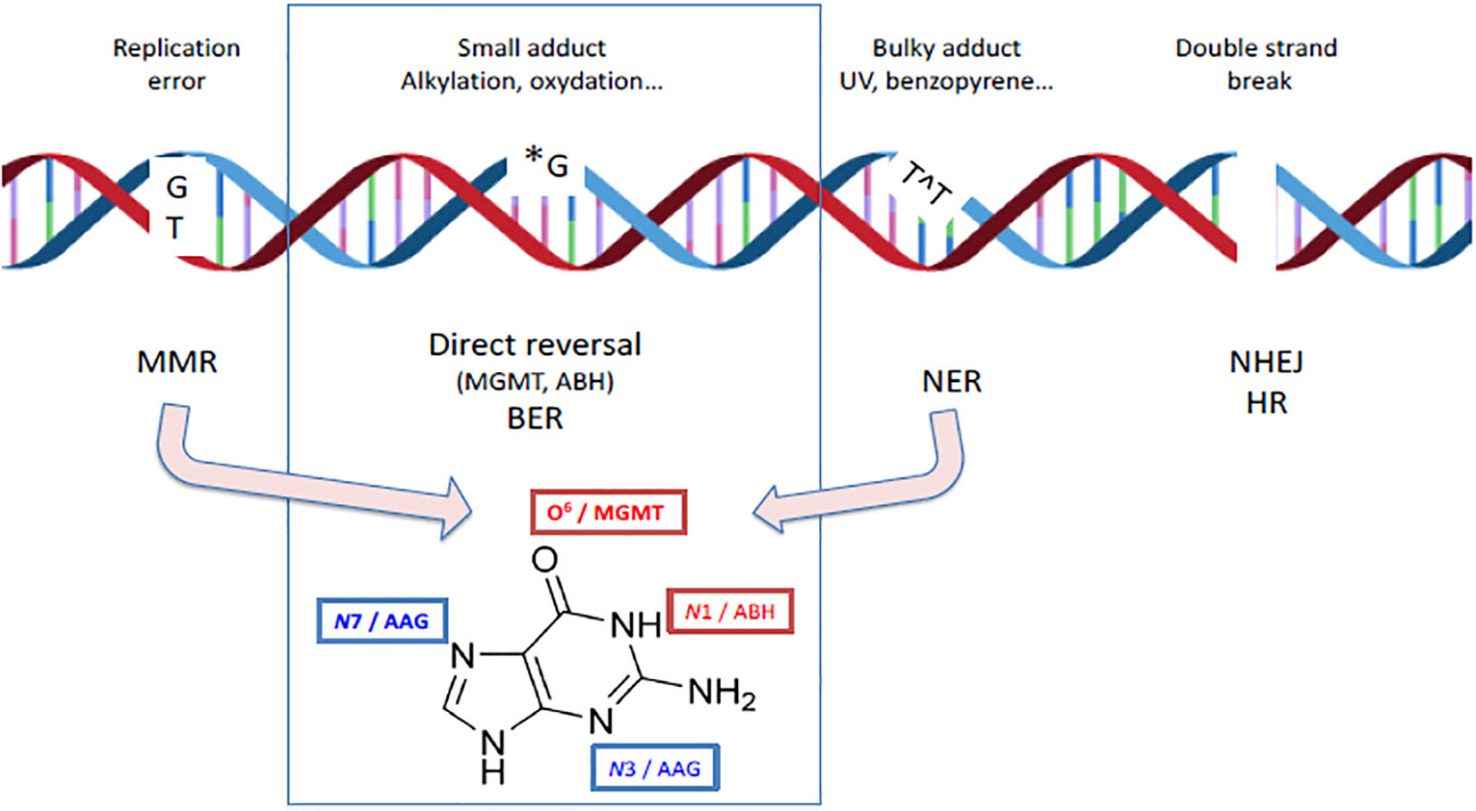
Schema of DNA damage types and corresponding repair pathways. The present paper focuses on alkylating damage: repair of alkyl lesions involves Direct Reversal (DR) pathways (*via* ABH (AlkB Homolog) or *via* methylguanine methyltransferase (MGMT)) and Base Excision Repair (BER; AAG glycosylase); the example of Guanine shows the positions repaired by Direct Reversal in red and the positions repaired by BER in blue. Pathways not classically involved in alkylating damage repair are shown to highlight overlap among pathways: for instance, mismatch repair (MMR) is involved in O^6^‐alkylguanine metabolism leading to toxic repair intermediates. Alkylating adducts bulkier than methyl groups can also be repaired by nucleotide excision repair (NER).

Chemical adducts in DNA have been shown to interfere with DNA replication and transcription. Under most circumstances, adducts are accurately repaired before DNA replication occurs. In such cases, there will be no genetic consequences as the DNA adducts are removed and faithfully repaired, and the DNA is restored to its original primary sequence. In contrast, replication of DNA containing adducts is likely to generate point mutations in the daughter strands at the site of adducts. Pathways that deal with the presence of lesions in DNA during replication are referred to as Lesion Tolerance pathways (Fuchs, 2016). These mechanisms are not genuine repair mechanisms, as they only allow replication to be completed despite the presence of lesions, rather than removing them. Figure 2 shows the main DNA repair pathways focusing on the ones pertinent to *N*7‐alkylG adducts and O^6^‐alkylG adducts.

A key determinant in potential effects of DNA adducts is whether or not the particular adduct blocks DNA replication. Replication‐blocking adducts transiently stop the replication machinery, inducing specific DNA damage responses that allow DNA repair to remove residual lesions genome‐wide; some adducts that do not block replication are thus potentially more mutagenic.

#### Adducts that Do Not Block Replication

The efficiency with which adducts that do not block replication are converted into mutations is highly variable depending on their chemical structure. Some adducts are converted into point mutations at near stoichiometric efficiency, while others exhibit low intrinsic mutagenic potency. Illustrative examples include two important alkylating adducts that are converted into point mutations with high (O^6^‐alkylG) and low (*N*7‐alkylG) efficiency, respectively. Neither *N*7‐alkylG nor O^6^‐alkylG adducts impede normal replication very much, thus do not block replication. Mutations triggered by these adducts are, thus, introduced into the genome by the normal replication machinery. During replication, O^6^‐alkylG adducts pair with thymine at high frequencies (>80%) instead of with cytosine, due to the disruption of normal hydrogen bond pairing caused by the adduct (see Fig. 1), thus generating G to A transitions. In contrast, *N*7‐alkylG adducts are converted into mutations at very low frequency, that is, below 0.1%. The intrinsic efficiency of conversion of these two alkyl guanine adducts differs by over three orders of magnitude (Philippin et al., 2014). It turns out that for adducts that exhibit high intrinsic efficiency of conversion into a mutation during replication, highly efficient, specific, and sometimes overlapping, repair pathways have been selected by evolution to repair them before replication in order to minimize mutagenesis during replication. This is the case for lesions such as *O* ^6^‐MG or 8‐oxo‐7,8‐dihydroguanine (8‐oxoG).

#### Replication‐Blocking Adducts

In contrast, there are many adducts that replication enzymes cannot bypass. In these cases, after transient pausing of the replicative polymerase, replication resumes downstream, owing to a re‐priming mechanism that leaves a gap containing the lesion. These gaps are repaired either accurately by a recombination mechanism that uses the sister chromatid to recover the missing information, or by Translesion Synthesis (TLS), an inherently error‐prone process. Indeed, TLS is mediated by low fidelity polymerases (TLS polymerases) that tolerate chemical modifications of the bases and are able to replicate past the lesion with a high probability of inserting an incorrect base. Among the many replication‐blocking lesions, AP sites, resulting from the hydrolysis of the *N*‐glycosidic bond between the N9 of guanine and the C1 of 2‐deoxyribose, are perhaps the most common. The rate of AP site formation is low at normal G residues (3 × 10^−11^ sec^−1^ at 37°C) but increases strongly (over 10^5^‐fold) by *N*7 alkylation (1.4 × 10^−6^) (Gates et al., 2004). AP sites are normally repaired by base excision repair (BER) with AP endonuclease action as a first step. When AP sites are present at the replication fork they strongly impair DNA synthesis and may elicit a cellular response, namely the TLS pathways. This entails the transient recruitment of specialized DNA polymerases that are able to insert a nucleotide across lesions such as AP sites. This process is intrinsically mutagenic, potentially leading to “indirect” mutagenesis by *N*7‐alkylG lesions.

### Overview of DNA Repair for *N*7‐ and O^6^‐alkylG LMW Adducts

As shown in Figure 2, there are several DNA repair pathways used by cells. The various repair pathways can be classified into two broad branches determined at the initial lesion recognition step. On one side, base repair pathways, either by direct reversal (DR) or by BER, exhibit a lesion recognition step that involves a protein with high affinity for one (or a few) lesions (narrow recognition specificity). In contrast, nucleotide excision repair (NER) exhibits broad substrate specificity that is achieved by several protein complexes that sense perturbations in the structure and/or dynamics of the DNA double helix induced by lesions without direct readout of the lesion itself. It is viewed more as a back‐up repair for alkylated DNA. *N*7‐ and O^6^‐alkylG adducts are known to be repaired by BER and DR, respectively (Barnes and Lindahl, 2004, Wyatt and Pittman, 2006).

#### Repair of N7‐alkylG Adducts

Alkylation adducts such as *N*7‐MG (and *N*3‐MA and *N*3‐MG) are repaired *via* the BER pathway that is initiated with recognition and excision of the modified base by a DNA glycosylase, followed by repair synthesis for sequence restoration. The recognition step is carried out by specific DNA glycosylases removing the damaged bases by hydrolyzing the *N*‐glycosidic bond (Kleihues and Margison, 1974). The glycosylase responsible for the repair and removal of *N*7‐alkylG and *N*3‐alkylA lesions is *N*‐methylpurine‐DNA glycosylase (MPG), also known as 3‐alkyladenine DNA glycosylase (AAG) or alkylpurine DNA *N*‐glycosylase (APNG) (Gupta et al., 1982, Swenberg et al., 1984). MPG is a homologue of the *E. coli* AlkA protein and is a monofunctional type I glycosylase (Randerath et al., 1985). Both down‐regulation and over‐expression of MPG sensitize cells to methylating agents (Phillips, 2005, Phillips and Arlt, 2007), suggesting that fine‐tuning of the expression levels of the enzymes involved in BER is essential for proper function *in vivo* (Xiao and Samson, 1993, Coquerelle et al., 1995, Glassner et al., 1998). The later steps of BER involve repair synthesis, mostly by DNA Pol β or Pol δ and subsequent ligation.

#### Repair of O^6^‐alkylG Adducts

O^6^‐MG (and O^4^‐MT) adducts are subject to DR mediated by methyltransferases (also referred to as ATase and AGT). These proteins repair O^6^‐alkylG adducts in a one‐step, irreversible reaction that transfers the alkyl group from the O^6^‐oxygen in the DNA to a cysteine residue in the reactive pocket of methylguanine methyltransferase (MGMT), thereby restoring the DNA sequence and, in turn, inactivating the MGMT molecule. As one MGMT molecule can repair only one alkyl adduct, the cell's capacity for removing DNA O^6^‐alkylG adducts depends on the total number of MGMT molecules per cell and its rate of *de novo* synthesis (Kaina et al., 2007). Moreover, O^6^‐MG adducts are also targeted by Mismatch Repair (MMR) proteins leading to cytotoxicity (Klapacz et al., 2009).

### Endogenous Exposome

It has become clear in recent years that there exists an internal milieu called the endogenous exposome that comprises a multitude of endogenous reactive chemicals present ubiquitously in every living system investigated (Nakamura et al., 2014). These include metabolic products from intermediary metabolism, from normal defense mechanisms, and from disease processes; examples include ones produced by gut microflora, inflammation reaction products, and oxidative stress products, and ones from reaction to infections. It is thought that the ubiquitous endogenous DNA damage resulting from the endogenous reactive chemicals may drive background/spontaneous mutation processes. The most abundant endogenous DNA lesion is the AP site (Nakamura et al., 2000, Swenberg et al., 2011); these are highly mutagenic if present during DNA replication (Neto et al., 1992, Schaaper and Loeb, 1981). The next most abundant endogenous DNA lesions reported are *N*7‐HEG, *N*7‐(2‐oxoethyl)guanine (*N*7‐OEG), and 8‐oxoG (Boysen et al., 2010, Swenberg et al., 2011, Nakamura et al., 2014). These DNA oxidation products can result from lipid peroxidation products or reactive oxygen species (ROS), such as highly reactive hydroxyl radicals that are formed through a Fenton reaction involving hydrogen peroxide (H_2_O_2_), itself an abundant endogenous ROS. The most abundant endogenous DNA lesions after those mentioned include formaldehyde‐related *N* ^2^‐HOMdG and *N* ^6^‐HOMdA, acetaldehyde‐related *N* ^*2*^‐ethylidenedG and 1,*N* ^*2*^‐propano‐dG, and *N*7‐MG. Incorporation of available information on the endogenous exposome, the resulting endogenous DNA lesions, and their expected impact on risk, should be integrated with data on chemical‐specific adducts, especially where they are the same identical chemical entity.

## DEFINING A RESEARCH STRATEGY TO SUPPORT UNDERSTANDING THE APPROPRIATE ROLES FOR INDUCED LMW DNA ADDUCTS IN HAZARD AND RISK ASSESSMENT

Consideration of the overall consequence of these issues, stemming from the demonstrated presence of chemical‐specific DNA adducts, presented a substantial challenge to several chemical‐specific panels from the American Chemistry Council (ACC) and sector groups from the European Chemical Industry Association (Cefic). Each of these groups already had related work on‐going, with plans for more work in this arena. So, the industry groups agreed to work together in a loose coalition (Joint Industry Group; JIG^1^) to provide key data toward elucidation of the biological significance of DNA adducts, and their appropriate role in chemical risk assessment. Although not a formal organization, the JIG coalition shared information among the members and held several joint meetings to discuss research results and to co‐ordinate plans for further work. The coalition's focus was on LMW alkyl/hydroxyalkylG adducts, based on their chemical products (E, EO, P, and PO), which were either reactive epoxides (EO, PO) or could result in the formation of reactive epoxides (E, P), and had been demonstrated to result predominantly in the corresponding *N*7‐hydroxyalkylG DNA adducts (*N*7‐HEG, *N*7‐HPG). These efforts were formally launched in 2001 with a first workshop^2^ described in a published summary report (Pottenger et al., 2004).

This workshop was designed around three questions that seemed to highlight the unresolved issues:

- “What does an increase in chemical‐specific DNA adducts indicate?
- With particular reference to low levels of DNA adducts, what data demonstrate a consequence or otherwise for human health?
- What status should DNA adduct measurements have in overall hazard and risk assessment?”

As described in the publication, the invited academic experts debated these three questions and, from that debate, an overall strategy for data collection was developed to address the gaps identified. The projects were loosely defined and were to be conducted roughly in parallel, although some occurred sequentially; most were conducted using recognized DNA reactive “positive control” mutagenic chemicals (ethyl methane sulfonate (EMS), methyl methane sulfonate (MMS), ethyl nitrosourea (ENU), methyl nitrosourea (MNU)), and/or the chemicals of specific interest (EO and PO). The projects were grouped around three different approaches to the same question: is there a threshold for induction of a quantifiable level of mutation/genotoxic effects from these LMW, mostly *N*7‐guanine DNA adducts.

The first approach was the conduct of a series of ‘titration’ experiments, which compared dose–response curves for mutation induction (gene mutation and micronucleus (MN)) by low doses of MMS/MNU and EMS/ENU with *in vitro* assays.

The second approach was to structurally quantify induced DNA adducts and to quantify the resultant induction of mutation, using high‐power analytical techniques combined with a shuttle vector system for mutation detection. These experiments were conducted with adducts resulting from EO exposure.

The third approach utilized site‐specific mutagenesis, which included synthesis of DNA oligomers with chemical‐specific DNA adducts incorporated, and then testing of those adducted oligomers in a bacterial mutation detection system for their mutagenic potency. This permitted comparison between adducts formed by EO and PO (and their methyl counterparts) and unmodified guanine residues to determine their mutagenic potential.

### Approaches and Results

The aforementioned three specific research projects were supplemented with publication of a review on thresholds in genotoxicity (Jenkins et al., 2005), and research update meetings were convened regularly, culminating in research publications/presentations, and another workshop (Pottenger et al., 2009b). Additional related work was conducted by the researchers involved which, although not necessarily funded by JIG member groups, further extended and complemented the three main projects. Complementary research and other contributions from a variety of groups are also described later in this paper.

### Dose‐Response Analysis of Mutations and Chromosome Damage Induced by Direct‐Acting Alkylating Agents

**Figure 3:**
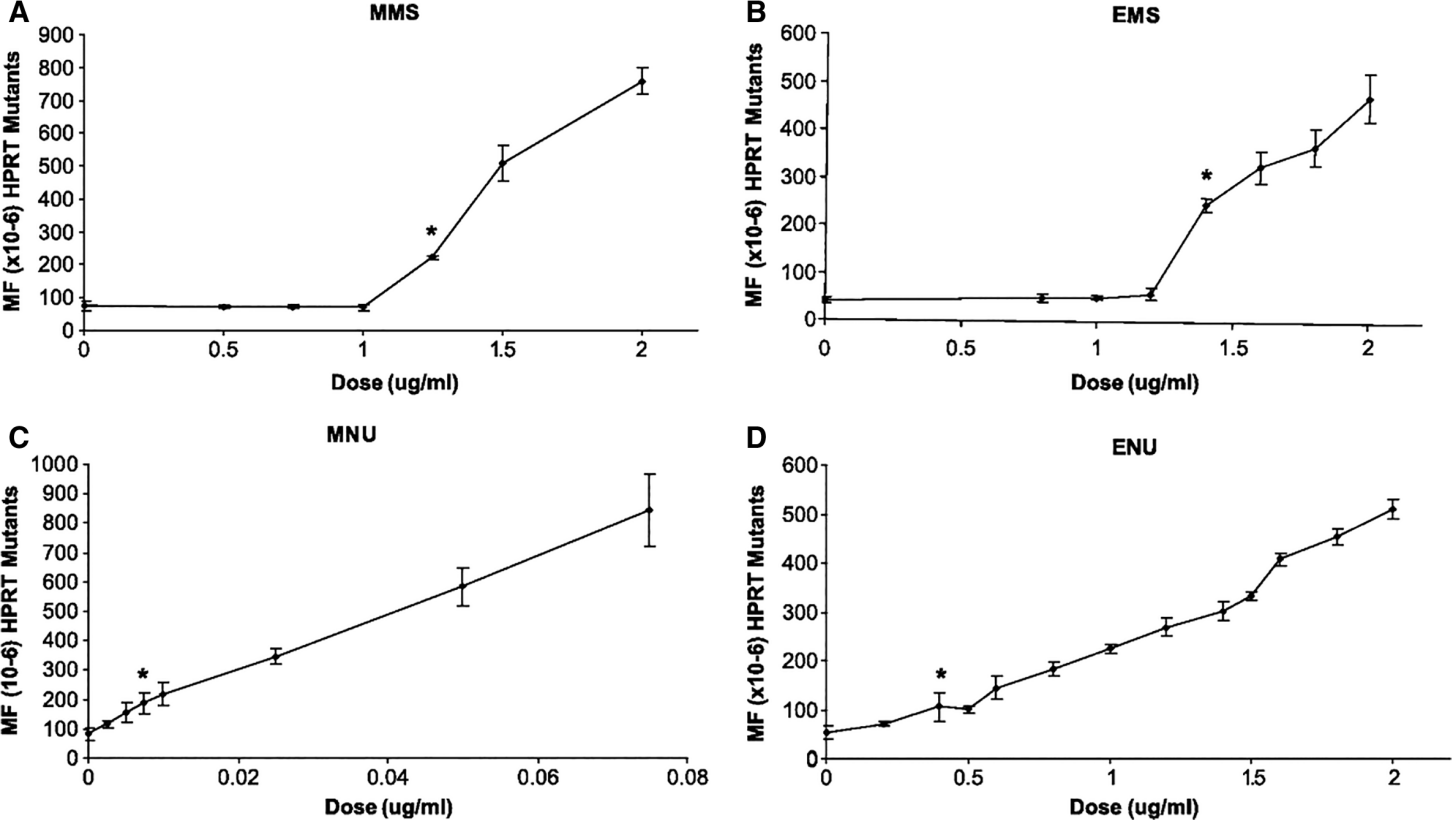
Reprinted (with permission) from Doak et al., 2007: “Figure 1. Influence of MMS (A), EMS (B), MNU (C), and ENU (D) dose upon micronucleus frequency in the AHH‐1cell line. Points, mean of treatments done in duplicate; bars, SD. *, the first statistically significant increases in chromosome damage at 0.85 μg/mL MMS (A), 1.40 μg/mL EMS (B), 0.15 μg/mL MNU (C), and 0.50 μg/mL ENU (D); %Mn/Bn, percentage of binucleated cells containing one or more micronuclei.”

Doak and colleagues (Doak et al., 2007) described *in vitro* gene mutation and chromosome damage dose–response data for MMS, MNU, EMS, and ENU. These were evaluated using the AHH‐1 human lymphoblastoid cell line and measuring responses with the hypoxanthine–guanine phosphoribosyl transferase (HPRT) forward mutation assay and the *in vitro* cytokinesis‐blocked micronucleus (MN) assay, all with a large number of cells and doses (*e.g*., 8–20 doses tested and up to 5‐fold more cells analyzed than typical for certain doses) tested to maximize the sensitivity of detection of a response. This dataset provided a detailed look at the dose–responses for induction of genotoxic effects by these DNA‐reactive chemicals. The resulting dose–response data demonstrated quantifiable no‐observed‐genotoxic‐effect‐levels (NOGELs) for MMS and EMS with both assays. However, while neither ENU nor MNU had doses with no increases, statistical NOGELs were defined in both assays (HPRT and MN) for both ENU and MNU (for example, see Fig. 3). These dose–response data were further analyzed using the bilinear/threshold dose–response modelling approach as described by Lutz and Lutz (2009), as published by Johnson et al. (2009). This dose–response modelling approach uses statistics to compare the fit of a dataset to a bilinear/threshold model with its fit to a linear model, thus allowing for a statistical determination of a better fit between these two models and, where appropriate, defining a Threshold dose (Td) value. The modelled results of the Doak et al. datasets indicated that the bilinear/threshold model provided a better fit to the MMS and EMS dose–response data both for gene mutations and MN induction, while the MNU and ENU data were mixed. ENU HPRT results were a better fit for the bilinear dose–response model, while the MN results for ENU were better described by a linear one; MNU results could not exclude a linear dose–response model for either endpoint.

**Figure 4:**
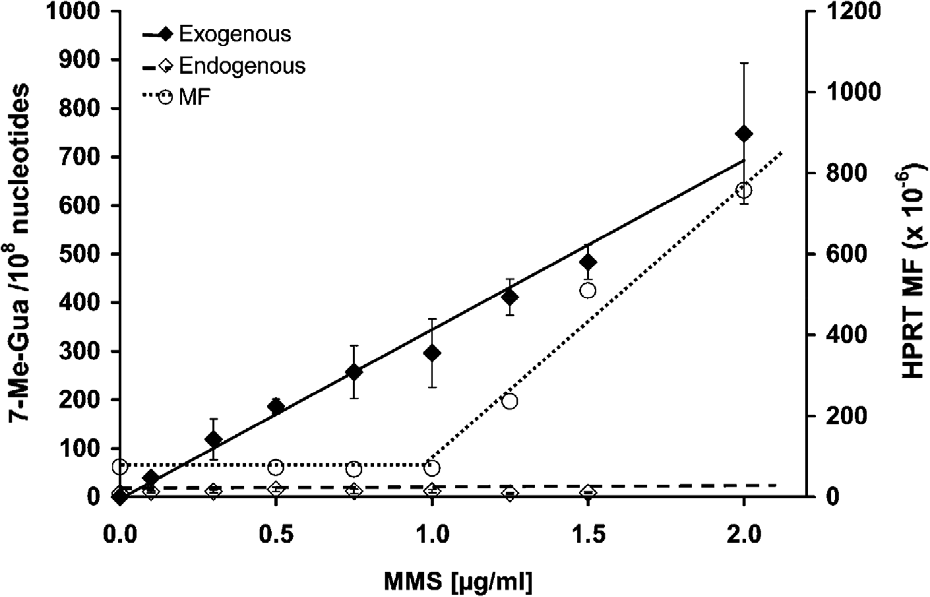
Reprinted (with permission) from Swenberg et al., 2008: “Figure 8. Comparison of N7‐methyl guanine DNA adducts and HPRT mutations in AHH‐1 cells exposed to MMS for 24 h. The endogenous adducts are N‐7Me‐G (⋄), while the exogenous adducts are [^13^C^2^H_3_]‐7Me‐G (♦). The Hprt mutant frequency is shown as (○).”

**Figure 5:**
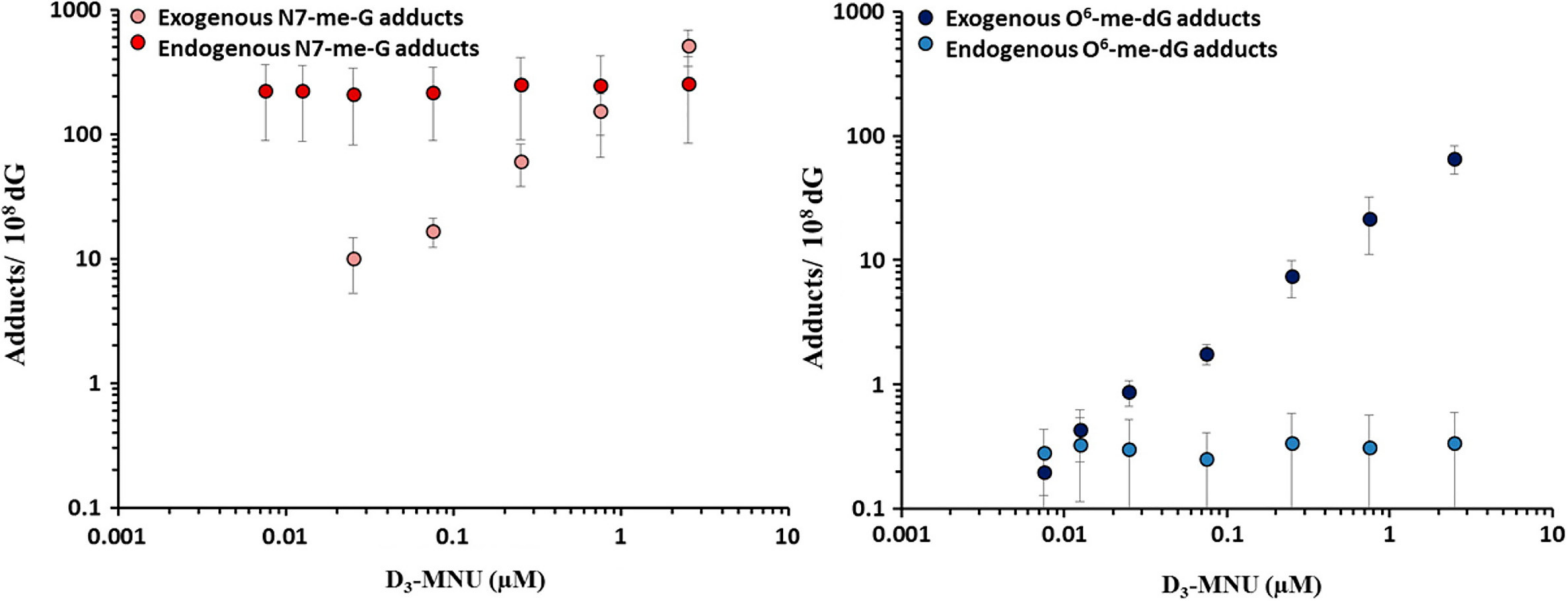
Reprinted (with permission) from Sharma et al., 2014: “Figure 1. Endogenous versus exogenous adducts in AHH‐1 cells exposed to D3‐MNU (0.0075 μM to 2.5 μM) for 1 h. The endogenous and exogenous O^6^‐me‐dG and N7‐me‐G adducts at each exposure concentration are plotted on a log versus log scale. Data represent the mean ± SD.”

Complementary work conducted by Swenberg and colleagues provided some adduct dose–response data to match the doses evaluated for genotoxic effects by Doak et al., (2007). Both endogenous and exogenously‐induced *N*7‐MG and O^6^‐MG were quantified in AHH‐1 cells treated with stable isotope‐ and deuterium‐labelled MMS ([^13^C^2^D_3_] MMS) or deuterium‐labelled MNU ([^2^D_3_]‐MNU) to increase the specificity and sensitivity for detection of these adducts and, importantly, to differentiate between endogenously present adducts (not labelled) and the exogenously induced ones (labelled). The data demonstrated a linear dose–response for labelled *N*7‐MG (both from MMS and from MNU), corresponding with exogenously induced *N*7‐MG adducts, while the endogenous *N*7‐MG did not increase over the 24‐h exposure to either MMS or MNU (Swenberg et al., 2008, Sharma et al., 2014) (see Figs. 4 and 5). A key finding from these data comes from comparison of the curves for exogenously induced adduct dose–response to the curves for mutation/genotoxic effect dose–response, where it becomes apparent that the shape of the mutation curves and the shape of the molecular dose of *N*7‐MG are quite different, most notably for MMS. The labelled *N*7‐MG adducts increase linearly over the dose range analyzed; however, the genotoxic effects (mutations, MN) reported by Doak et al. (2007) are nonlinear over that same dose range, with a low‐dose section not showing any corresponding increase in genotoxic effects (flat slope). Thus, increasing quantifiable levels of (exogenously induced) DNA adducts (lesions) did not correspond with an increase in genotoxic response. Together these datasets demonstrate that there are regions of the low‐dose dose–response where exogenously induced (labelled) *N*7‐MG adducts are increasing, but with no corresponding quantifiable increase in genotoxic response (Figs. 3 and 4). The *N*7‐MG adduct levels induced at the tested doses were not pro‐mutagenic (did not induce increased mutations), and therefore do not contribute to increases in genotoxic events such as mutations or induction of micronuclei. It is likely that other DNA lesions, ones that are pro‐mutagenic lesions such as the O^6^‐alkylG adducts, are the cause of the genotoxic events. Those pro‐mutagenic lesions comprise a smaller proportion of total adducts and may not even be present at such low doses. The adduct profile data published by Beranek, (1990) would support this hypothesis, as O^6^‐MG represents only a small proportion of the adduct profiles for all four of these mutagenic chemicals (from not detected to 16.6% in cultured cells and in mammalian liver).

The *in vitro* experimental work in AHH‐1 cells was then extended at Swansea University, evaluating mutation dose–response for lower doses, and using DNA repair knock‐down techniques to manipulate the cell's repair capacity for more mechanistic analyses. For example, the DNA repair protein MGMT, which repairs the pro‐mutagenic O^6^‐alkyl‐G adduct by removing the alkyl group, was pre‐depleted with O^6^‐benzylguanine pretreatment. The resulting reduction in DNA repair capacity led to a modified *HPRT* gene mutation dose–response curve following exposure to MNU. The dose–response curve for genotoxic effects in cells with pre‐depleted repair was non‐linear (J‐shaped), substantiating the existence of NOGELs for point mutation induction by direct‐acting alkylating agents. The shifted dose–response curve led to determination of a lower POD (NOGEL) for MNU under these conditions compared to cells with normal MGMT capacity (Thomas et al., 2013). A different mechanistic approach manipulated the levels of the DNA repair enzyme MPG, which permanently removes the alkylated base from the DNA backbone as the first step in BER. This reduction in MPG affected the dose–response for EMS‐induced MN, as the MN POD moved to a lower value after MPG was knocked‐down using a specific RNAi (Zaïr et al., 2011, Thomas et al., 2013). The cells with diminished DNA repair capacity demonstrated decreased POD values compared to similarly treated cells with normal DNA repair capacity. Therefore, POD metrics (*e.g.,* NOGELs) can be defined for the potent alkylnitrosoureas, MNU and ENU, and when DNA repair is knocked‐down the POD value decreases.

The extensive genotoxicity dose–response data from Doak et al. (2007) were further analyzed, along with other examples of similar dose–response datasets, using a wider toolbox of approaches to define POD metrics described later on (Gollapudi et al., 2013, Johnson et al., 2014). Clear benchmark dose (BMD; BMDL) metrics were derived for each of these dose–response datasets, and bilinear/threshold model‐derived Break Point Doses (BPD/L) were identified for EMS and MMS, based on the Doak et al. (2007) data; analysis of these data for ENU and MNU did not identify BPD/L values.

Overall, the *in vitro* dose–response “titration” data of dose *versus* mutations demonstrated that threshold values or BPDs defined using bilinear/threshold models can be identified for genotoxic effects (mutation/MN) caused by DNA‐reactive chemicals. The additional work conducted by the Swansea and Swenberg labs provided data indicating that the *N*7‐alkylG adducts were not likely to be the cause of these genotoxic effects, along with some mechanistic data to explain why these low‐dose regions demonstrate no quantifiable increases in genotoxic effects, informing the MOA for a NOGEL, even with direct‐acting mutagens.

### Mutagenic Effects Induced by Exposure to Ethylene Oxide

EO is a widely used industrial intermediate in the manufacture of chemicals; it is also employed as an agricultural fumigant and as a sterilizing agent, although these are minor uses. EO is classified by the International Agency for Research on Cancer (IARC) as carcinogenic to humans, despite conflicting epidemiology data regarding the ability of EO to induce specific cancers or increase cancer‐related mortality (IARC, 1994, 2008). Moreover, a confounding factor in evaluating the risks associated with occupational or environmental exposure to EO is the fact that ethylene is generated *in vivo* during normal physiological processes and can be converted to the epoxide by cytochrome P450 2E1‐mediated metabolism (IARC, 1994, 2008). Consequently, humans are constantly exposed to EO, and this is reflected by the fact that *N*7‐HEG adducts are detectable in isolated lymphocytes from people who were not knowingly exposed to EO (Zhao and Hemminki, 2002, Yong et al., 2007).

**Figure 6:**
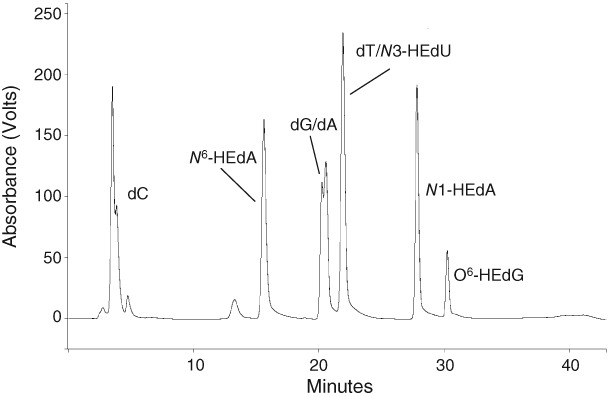
Reprinted (with permission) from Tompkins et al., 2008. “Figure 4. HPLC chromatogram demonstrating the separation of hydroxyethyl (HE) nucleoside adducts from unmodified nucleosides, using the conditions employed for the isolation of nucleoside‐HE adducts. Synthetic adduct standards have been used to produce this trace for illustrative purposes; in a typical digested DNA sample HE adducts would not usually be present at detectable levels and fractions would be collected on the basis of retention time.”

In order to better understand the significance of DNA damage induced by EO, and any resulting mutations, it is important to ascertain which specific EO adducts have mutagenic potential (are pro‐mutagenic), and to perform studies at doses and damage levels that are relevant to human exposures. When EO reacts with DNA, the most abundant product formed, *N*7‐HEG, is not thought to be directly pro‐mutagenic; however, it readily depurinates leaving AP sites that have miscoding potential if not repaired before replication occurs (Takeshita et al., 1987). Minor amounts of *N*3‐(2‐hydroxyethyl)adenine (*N*3‐HEA) and O^6^‐HEG have also been identified from this interaction *in vitro*, while 2‐hydroxyethylation can also occur at the *N*1 and *N* ^6^ positions of adenine (Tompkins et al., 2008). The *supF* forward mutation assay, a shuttle vector assay, has been used to investigate the biological relevance of low levels of EO‐induced DNA adducts in human cells. The aim was to determine whether there is a level of tolerable DNA damage, below which notable increases in mutations are not induced; in addition, extensive analytical work would begin to address the specific role of different HE‐DNA adducts (Tompkins et al., 2009). This assay involves *in chemico* treatment of a shuttle vector plasmid carrying the *supF* gene, which is then transfected and replicated in human cells in culture followed by recovery and screening for the presence of mutations in *E. coli*. Any plasmids with a mutated s*upF* gene can then be characterized by sequencing, thereby providing a quantitative assessment of mutagenic potency for the compound of interest and a mutation spectrum. A major advantage of this approach is the ability to profile the type and level of damage present on the plasmid prior to transfection into human cells; this quantification was conducted for EO using a validated liquid chromatography–tandem mass spectrometry (LC–MS/MS) assay capable of detecting five different HE‐adducts (see Fig. 6) (Tompkins et al., 2008). In an initial study, exposure to “low” concentrations of EO (0.01 to 2 mM) generated levels of *N7*‐HEG in the plasmid that far exceeded those typically detected in human DNA, but no other HE‐lesions were detectable. This level of damage failed to produce a notable increase in mutation frequency in *supF* above background rates when replicated in cells. The mutation spectrum was not different from the spontaneous distribution, indicating that the *N*7‐HEG adduct did not result in quantifiable increases in mutation with the *supF* assay, despite the heavy *N7*‐HEG adduct load. Thus, EO is not strongly mutagenic in this system and *N7*‐HEG itself has low mutagenic potential (Tompkins et al., 2009).

It took much higher EO concentrations (10 mM EO for 24 h) to induce any quantifiable increase in mutation frequency. At this very high dose, the minor lesions *N*1‐HEdA, O^6^‐HEdG, and *N*3‐HEdU were detected in treated plasmid along with high levels of *N7*‐HEdG. These findings imply that either a certain level of total adducts or specific pro‐mutagenic adducts must be present before mutations become evident above background. Based on comparison of these results with published data for other genotoxic compounds analyzed in the *supF* forward mutation system, it was concluded that EO is a relatively weak mutagen in human cells.

**Figure 7:**
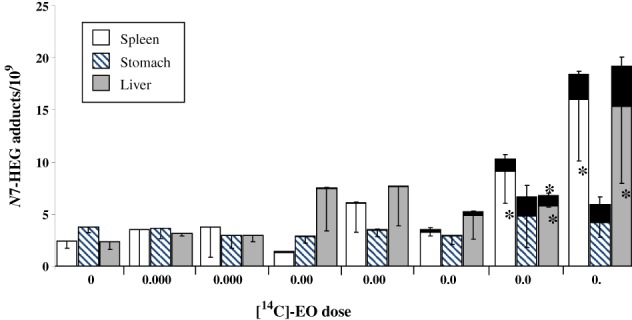
Reprinted (with permission) from Marsden et al.*,* 2009. “Figure 2. Contribution of endogenously and exogenously derived N7‐HEG to the total adduct level in tissues of [^14^C]EO‐treated rats. Endogenous adducts were determined by LC–MS/MS. Exogenous ^14^C‐labelled adducts were quantified by AMS and are shown as black bars on top of bars representing endogenous adduct levels. Columns, mean of three animals per group; bars, SD. *, P < 0.05, the level of endogenous N7‐HEG in tissues of [^14^C]EO‐treated rats is significantly higher than the corresponding background level in control animals; **, P < 0.05, the total level of adducts (endogenous plus exogenous) is significantly higher than the level of endogenous adducts alone in a particular tissue.”

Given that EO is produced in humans from the metabolism of ethylene generated during physiological processes, it is essential to determine the relative contribution of the ever present endogenous versus exogenously derived DNA damage and its consequence, to allow for more accurate human risk assessments. This became possible through the use of a dual‐isotope approach that enabled delineation of the *in vivo* dose–response relationships in rats over a concentration range relevant to human EO exposures, including doses associated with occupational sources (Marsden et al., 2009). Although this project was funded separately by the ACC Long Range Initiative (LRI) and was not part of the JIG research efforts, it is described here since the findings are integral to understanding the role of EO‐induced damage and any associated risks. By combining LC–MS/MS analysis and the extremely sensitive technique of HPLC‐accelerator mass spectrometry (AMS), both the endogenous and exogenous ([^14^C]‐labelled) *N*7‐HEG adducts were quantified in tissues of [^14^C]‐EO‐treated (i.p. injection) rats (Marsden et al., 2007, 2009). Although levels of [^14^C]‐*N*7‐HEG induced in DNA extracted from treated rat tissues increased in a quasi‐linear manner, the level of adducts arising *via* this route was negligible compared to the much higher natural background abundance of endogenous *N*7‐HEG present (see Fig. 7). The implications of this result are that exogenous *N*7‐HEG formation may not pose any additional risk over and above that presented by the ubiquitous background damage, at least up to the tested (high, i.p.) doses. It is worth noting that, at the two highest doses studied (0.05 and 0.1 mg EO/kg bw, i.p. injection), administration of [^14^C]‐EO substantially increased the endogenous *N*7‐HEG levels in the liver and spleen of treated rats, suggesting EO can induce physiological pathways responsible for ethylene generation *in vivo* and thereby indirectly promote endogenous *N*7‐HEG production.

Overall, the experimental data demonstrate that EO is only weakly mutagenic in the *supF* system, as quantifiable increases were only observed following exposure to high concentrations, and that EO‐induced (exogenous) *N*7‐HEG adduct levels are overwhelmed by endogenous *N*7‐HEG adduct levels across a range of EO doses (i.p. injection).

### Mutagenic Response to Site‐specifically Modified DNA Probes

The goal of this JIG project was to test the *N*7‐alkyl/hydroxyalkylguanine adducts of interest specifically for their capability to induce a mutagenic response, using site‐specific mutagenesis techniques. This direct test of their mutagenic potential seemed a logical next step, although it posed many challenges. The first challenge was the instability of *N*7‐alkyl/hydroxyalkylG adducts as discussed previously, and whether synthesized adducted oligomers were stable enough to test. Thus, the decision was to test the O^6^‐alkylG adducts first, as a positive control and a good first proof‐of‐principle. O^6^‐alkylG adducts are considered pro‐mutagenic and so should induce mutations in the bacterial detection system.

#### Synthesis and Purification of Single‐adducted Oligonucleotides

Two main approaches exist for the preparation of DNA probes with O^6^‐ and *N*7‐HEG/HPG adducts, taking into account the well‐documented instability of *N*7‐alkylG adducts.

##### Site‐specific synthesis of O^6^‐HEG containing oligonucleotides

The chemical synthesis of oligonucleotides that contain any of the three O^6^‐alkylG adducts including O^6^‐HEG, and the two HPG isomers: O^6^‐(2‐hydroxypropyl)‐2′‐deoxyguanosine and O^6^‐(2‐hydroxy‐(1‐methyl)‐ethyl)‐2′‐deoxyguanosine, was achieved by adapting the well documented solid‐phase phosphoramidite method (Mazon et al., 2009). Thus, a convenient, O^6^‐protected, building block was synthesized and site‐specifically inserted into defined sequence oligonucleotides. Activation of the modified guanine moiety allowed a specific reaction with hydroxyethanol, 1‐hydroxypropanol, or 2‐hydroxypropanol, to generate O^6^‐HEG and the PO‐derived isomers: O^6^‐1HPG, and O^6^‐2HPG, respectively.

##### Preparation of N7‐(2‐hydroxyalkyl)‐guanine containing DNA fragments

However, the above synthetic method was not appropriate for the preparation of the *N*7‐alkyG containing oligonucleotides due to the instability of both the *N*‐glycosidic bond and the potential for opening of the imidazole ring of the 7‐substituted guanine 2′‐deoxyribonucleosides. Therefore, a less flexible alternative strategy was applied. A 13‐mer DNA fragment with only one guanine residue was treated with an excess of EO or PO and the main alkylation products were purified by HPLC. Thus oligonucleotides containing *N*7‐HEG and *N*7‐HPG were prepared (Philippin et al., 2014). These lesions were stable in isolated DNA when left in aqueous solutions at RT up to several days (Gasparutto et al., 2005). The lack of mutagenicity discussed below (Philippin et al., 2014) indicated a lack of detectable formation of the highly mutagenic AP sites or the pro‐mutagenic formamidopyrimidine ring‐opened derivatives from the *N*7‐G adducts (within the time frame of the experiment). Any more direct proof of the apparent lack of any formation of N^5^‐hydroxyalkyl‐FAPy adducts in both isolated and cellular DNA awaits further targeted measurement experiments, for example with an LC–MS/MS assay that allows the unambiguous detection of non‐substituted FapyG, an oxidatively generated guanine lesion (Cadet et al., 2010). This could be eventually accompanied by the mutagenic assessment of N^5^‐hydroxyalkyl‐FAPy lesions using a similar strategy that was applied for related *N*7‐substituted guanine adducts.

#### Repair of N7‐ and O^6^‐alkyl/hydroxyalkylguanine Adducts: Distinct and Novel Repair Strategies to Remove DNA Adducts

As discussed earlier, *N*7‐alkylG and O^6^‐alkylG adducts use different repair strategies, with *N*7‐alkylG adducts relying mainly on BER, often following depurination (Gates et al., 2004, Boysen et al., 2009), while the O^6^‐alkylG is repaired *via* DR with the removal of the alkyl group by MGMT. Interestingly, during the course of this project, a novel pathway that bridges BER and NER was identified (Mazon et al., 2009, 2010). As the size of the alkyl group increases, the repair of O^6^‐alkylG adducts appears to default to NER (Kaina et al., 1983, Margison et al., 2007, Tubbs et al., 2009, Aramini et al., 2010). Although not described here in any detail as tangential to the focus of this review, it seems this observation represents a novel paradigm showing that proteins with high binding affinity to specific lesions may be instrumental to channel processing of specific lesions from BER to NER, thus showing the key role played by the size of the O^6^‐substituent in the selection of the repair mode.

#### Tolerance of N7‐ and O^6^‐alkylG Adducts During Replication in Whole Cells/Assessment of their Intrinsic Mutagenic Potential

In order to assess how these adducts are processed during replication, a double‐stranded plasmid vector containing a single *N*7‐ or O^6^‐alkylG adduct was constructed (Mazon et al., 2010, Philippin et al., 2014). The main focus of the work was to determine the mutagenic potential of adducts formed by EO and PO, compared to their methyl and unmodified counterparts. *N*7*‐*alkylG adducts, such as *N*7‐HEG and *N*7‐HPG, have never been specifically tested previously, likely due to lack of stability from rapid depurination. To assess the potential effect of these adducts on DNA polymerase bypass activity we measured the effect of these adducts on replication in bacteria. In order to achieve maximal sensitivity single‐stranded plasmid probes carrying a single lesion were used. Under these very stringent conditions, colonies can only arise after successful replication across the single adduct. In addition, single‐stranded plasmids are particularly stringent probes for replication, as adducts cannot be removed by repair. The *N*7‐MG adduct did not impair replication, while the *N*7‐HEG and *N*7‐HPG adducts reduced the transformation efficiency substantially, to ≈70% and 40% relative to the unmodified construct, respectively. Interestingly, no significant loss in transformation efficiency was observed with any of the O^6^‐alkylG adduct carrying probes relative to the unmodified construct, indicating that these pro‐mutagenic adducts exhibit no (or only weak) replication‐blocking potential. Taken together, when compared to major replication‐blocking lesions such as UV‐induced pyrimidine dimer lesions that cannot be bypassed by the normal replication, but require the TLS machinery instead, neither the *N*7‐ nor the O^6^‐alkylG adducts severely affect normal replication.

#### Lack of N7‐alkylG Adduct‐induced Mutagenesis

To investigate the intrinsic mutagenic potential of *N*7‐ and O^6^‐alkylG adducts, single‐stranded DNA probes containing single adducts were evaluated. As stated previously, the O^6^‐alkylG adducts, considered as pro‐mutagenic, were tested first as a proof‐of‐principal approach (Mazon et al., 2009, 2010). As O^6^‐alkylG may potentially be repaired by ATase‐mediated reversal, even when present in single‐stranded DNA, the mutant fraction was assessed in an ATase‐deficient *E. coli* strain (*ada ogt mutS*). Under these stringent conditions, as adducts can neither be repaired by alkyl transfer nor by NER, plasmid progeny production necessarily relies on replication through the single adduct. All O^6^‐alkylG adducts (including both O^6^‐HEG and O^6^‐HPG) were highly mutagenic (mutant fraction >80%), implying that correct insertion of a C across the O^6^‐alkylG adducts is a minor event (<20%). As expected from reports on O^6^‐MG mutagenesis, sequencing of the mutants obtained with the different adducts under investigation demonstrated that they resulted from mis‐insertion of T across from O^6^‐alkylG, leading to G → A transition mutations (Bartsch, 1996, van Zeeland, 1996, De Bont and van Larebeke, 2004, Rundle, 2006). In the repair proficient wild‐type (WT) strain, O^6^‐MG‐induced mutagenesis is lost due to efficient ATase‐mediated repair. In sharp contrast, the hydroxypropyl adducts (O^6^‐1HPG and O^6^‐2HPG) remained as mutagenic in the WT strain as in the repair defective *ada ogt* strain, indicating no or only limited repair *via* DR. With the O^6^‐HEG adduct, a partial reduction of the mutant fraction was observed in the WT strain compared to the *ada ogt* strain, illustrating moderate repair of this adduct by the WT ATase proteins.

The results for the *N*7‐alkyl/hydroxyalkylG adducts were in sharp contrast to the above. Prior to evaluating the *N*7‐alkylG adducts, the presence of depurinated molecules was minimized. Following introduction of these probes into *E. coli* cells, the effect of the *N*7‐alkylG adducts on the fidelity of replication was determined. Within the detection limit of the assay, replication across the *N*7‐alkylG adducts *in vivo* (in bacterial cells) was essentially error‐free, as no mutant colony was observed among ≈1000 individual sequenced colonies (i.e., mutation frequency < 0.1%) (Philippin et al., 2014). Given these unprecedented results, the experiment was repeated with the exact same outcome—no mutations detected from any of the *N*7‐alkylG adducts tested. Thus, the completely reproducible results demonstrated a lack of mutagenicity for these *N*7‐alkyl/hydroxyalkylG adducts, including *N*7‐HEG and *N*7‐HPG. This is the first such demonstration of a lack of mutagenicity for LMW *N7*‐alkyl/hydroxyalkylG adducts.

### JIG Research Conclusions

In summary, the research program sponsored by JIG provided substantial data to address questions on dose–response relationships for adduct formation, mutation induction, and the relationship between some specific adducts, including chemical‐specific adducts that are derived from both endogenous and exogenous sources, and mutation induction. In all cases, the data demonstrate that these relationships can be non‐linear, with the LMW *N*7‐alkylG adducts (*N*7‐HEG, *N*7‐HPG) proving not to be mutagenic when specifically tested in site‐specific mutagenesis assays.

As a follow‐up to the aforementioned workshop that launched the JIG effort, a second workshop was held in September 2008 as a satellite of the EEMGS (European Environmental Mutagen and Genomics Society) annual meeting, which included reviewing the recent data against the original three questions. It was jointly sponsored by ECETOC (European Center for EcoToxicology), ILSI/HESI, and JIG. An overview of that follow‐up workshop, which included participation from the scientists who developed the data described above, along with other academic, industry, and regulatory scientists, and some regulators from Europe and the US, was published to share the findings (Pottenger et al., 2009b). The program comprised presentations of technical data and discussions around regulatory applications and implications, along with break‐out sessions on specific questions. Several consensus statements were developed at the end of the workshop, including ones addressing the following:

“DNA adducts should be considered as biomarkers of exposure, which may play a key role in establishing a mode of action (MOA) for cancer. Adducts themselves should not be considered as equivalent to mutations or later stage events in carcinogenesis. Although it was not possible at this time to agree on a general level of adducts below which there is no adverse biological effect, there are examples of genotoxic mutagens/carcinogens for which bilinear/“thresholds” have been demonstrated^3^. Evidence regarding “thresholds” for mutations should be considered on a case‐by‐case basis, in light of available MOA and mechanistic data, to build a knowledge base.”

Workshop participants agreed that it would be useful to develop guidance on a recommended format for data presentation (especially agreement on units and appropriate statistical analyses) — something that has been addressed since by the ILSI/HESI group as discussed later. Finally, a recommendation was made that early cases needed data to support a mechanistic explanation for any hypothesis of a “threshold” for mutations; this was considered essential to support the eventual use of this information in risk assessment.

## DISCUSSION: IMPLICATIONS, INTEGRATION, AND APPLICATIONS OF THE RESULTS AND COMPLEMENTARY EFFORTS

As the data and work products described above were being developed and became available, it was important to integrate the new approaches and new information into the larger picture, with the many, separate, contemporaneous efforts contributing to the debate. There were substantial efforts to address these questions by many researchers collecting similar data, with *in vitro* and *in vivo* models. Some work was directly complementary, other work was serendipitously independently corroborative. In addition, the new data were integrated with work products from international committees and with ideas brought forward from other workshops addressing interpretation and methods, all aimed at incorporation and acceptance of the learnings into MOA analysis and risk assessment—the ultimate goal.

### Implications from JIG‐sponsored Work

**Figure 8:**
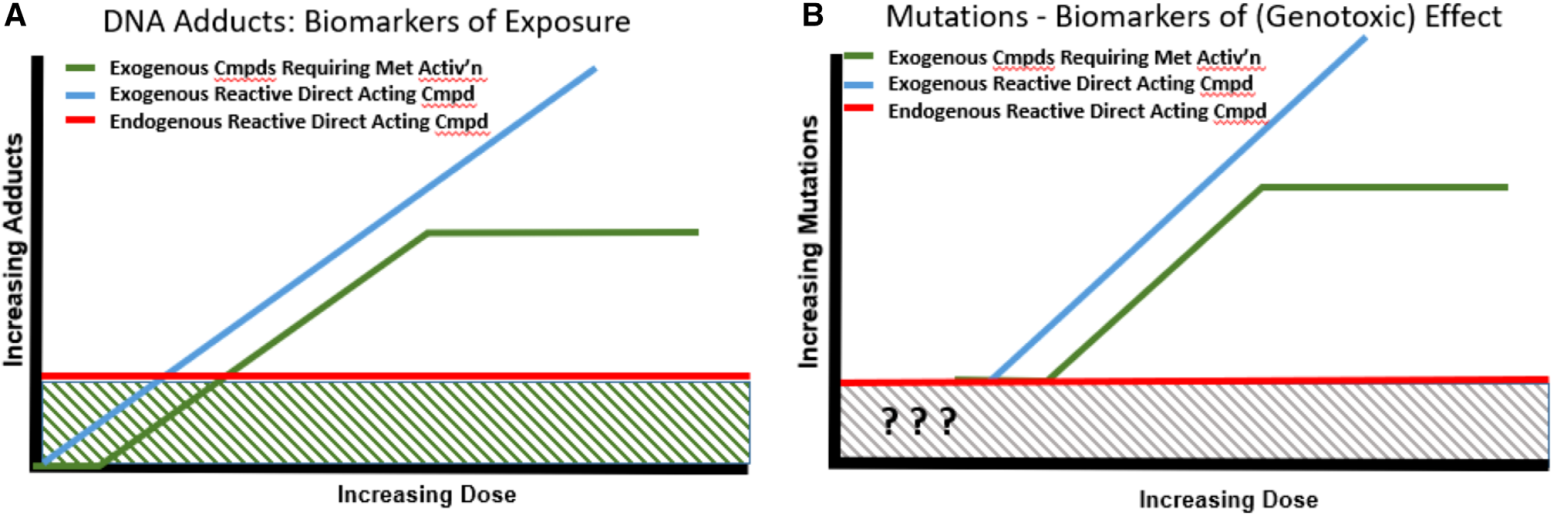
Schematic representation of theoretical dose–response curves for Biomarkers of (A) Exposure (DNA adducts) and (B) (Genotoxic) Effect (Mutations). Hashed regions represent background levels. 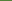 Exogenous Compounds Requiring Metabolic Activation. 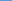 Exogenous Reactive Direct‐Acting Compounds. 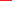 Endogenous Reactive Direct‐Acting Compounds.

The ground‐breaking analytical work built on Lindahl's much earlier efforts (Lindahl, 1993), with both stable isotopes and radioisotopes identifying and quantifying the many endogenously present DNA adducts. These included pro‐mutagenic adducts and adducts that are chemically identical to specific chemical‐induced ones, all found in tissues from untreated control animals under steady‐state conditions as described earlier (Swenberg et al., 2008, 2011, Nakamura et al., 2014). The growing database on endogenously present adducts has opened new avenues of perspective, leading to a paradigm shift that is still playing out in recognizing the impact of this information on background issues and on risk assessment. Some of the challenges are depicted in Figure 8. The idea that there is a background of a variety of DNA adducts (shown as green hashed region in Fig. 8A) ubiquitously present, offered a possible explanation for the well‐recognized background of spontaneous mutations (shown as grey hashed background in Fig. 8B), quantified in every properly controlled mutagenicity experiment for decades. The spontaneous/background mutations are thought to be due to incomplete or inadequate DNA repair of the background/endogenous DNA adducts. Those background/endogenous adducts derive from many sources, including endogenous reactive species formed during cellular processes, metabolism of DNA, *etc*. The ubiquitous endogenous background for adducts (Fig. 8A) and for mutations (Fig. 8B) render depiction of dose–response below the background a challenge—one that is overcome for the adduct dose–response by employing the specific and sensitive techniques with stable/radio isotope‐labelled chemicals described above. Thus, it is possible to track chemical‐specific adduct levels below the endogenous background, where one predicts a linear decrease to the starting point; this can be shifted due to *e.g*., required metabolism to reactive forms (green line in Fig. 8A). It is important to recognize a critical difference in information available from adducts compared to mutations—that chemical‐specific DNA adducts can be linked to a specific chemical exposure, because they retain chemical‐specific information related to their source. It may require sophisticated techniques to distinguish endogenous from exogenous chemical‐specific adducts, but it is feasible to do so.

On the other hand, mutations do not retain chemical‐specific information, thus it is impossible to demonstrate unequivocally the source of a specific mutation. There is no specific analogous analytical marker retained with a mutation that links them to a specific exogenous chemical exposure. However, in some cases, sequencing of mutations can tie them to a specific class of mutation that may be linked with a specific exposure. Aflatoxin B1 and G:C ➔T:A transversions in codon 249 of *p53* provide such an example (Moore et al., 2018). For the most part, it is not possible to determine the source (*e.g*., endogenous *vs* exogenous, and if exogenous, what exposure) of a mutation in the region below the endogenous background level, depicted in Figure 8B with question marks. Thus, a DNA adduct represents a biomarker of exposure, one that can be linked to a specific chemical exposure, while a mutation, as a heritable change in the primary DNA sequence, represents a biomarker of genotoxic effect.

Of course, although this discussion is focused on adducts and mutations, expression of a mutation requires more than just an adduct. Cell proliferation to fix the mutation is just one critical step in an MOA for mutation induction (Pottenger and Gollapudi, 2010). Indeed, tissue‐specific and cell‐specific proliferation rates have been shown to drive differences in tissue mutation rates (Long et al., 2018). Once the mutant frequency rises above the endogenous background level (depicted with the grey hashed lines), it generally increases until either the rate plateaus due to metabolic saturation of formation of the reactive metabolites responsible for the mutations, or it increases theoretically until cell death from toxic damage. However, understanding the dose–response for these adducts, whether endogenously present or exogenously induced, and how they relate to the possible outcome of induced mutation (above the background or spontaneous level), is a key aspect of this work. Although it is impossible to determine the dose–response for induced mutations within the region of background/spontaneous mutations, the important information for risk assessment is understanding at what exposures this genotoxic response surpasses the background/spontaneous level.

### Complementary Efforts

Substantial complementary research efforts have generated many published datasets, including some extensive ones, to measure mutation dose–response curves and, in several cases, incorporating exposure biomarkers of internal dose and dose–response. Key results from several research groups, including both *in vitro* and *in vivo* work, are briefly described below.

The dose–responses for genotoxic effects from a series of DNA‐reactive chemicals were evaluated *in vitro* for induction of micronucleus with flow cytometry measurements, and large numbers of doses and cells were evaluated to strengthen the statistical evaluations; these included vinblastine sulfate (VB), EMS, MMS, ENU, MNU, and bleomycin (BLEO) (Bryce et al., 2010). The authors concluded that all the test chemicals except BLEO had dose–responses that demonstrated a better statistical fit with a nonlinear dose–response model than a linear one. Similar *in vitro* dose–response data for induction of gene mutation (TK) in mouse lymphoma cells by DNA‐reactive chemicals MMS and MNU demonstrated a better statistical fit for a nonlinear model for MMS results, while the MNU results did not show a better fit for the nonlinear model (Pottenger et al., 2009a). These results are similar to the results Doak and colleagues (Doak et al., 2007) published for MMS/EMS and MNU/ENU as described above; the early data for alkylnitrosoureas appeared to fit a linear model better than a non‐linear one. However, further work with MNU showed that it was more a question of how low a dose was tested, as Thomas and coworkers (Thomas et al., 2013) later demonstrated that MNU exhibited a non‐linear dose–response, based on additional data collected at lower doses.

**Figure 9:**
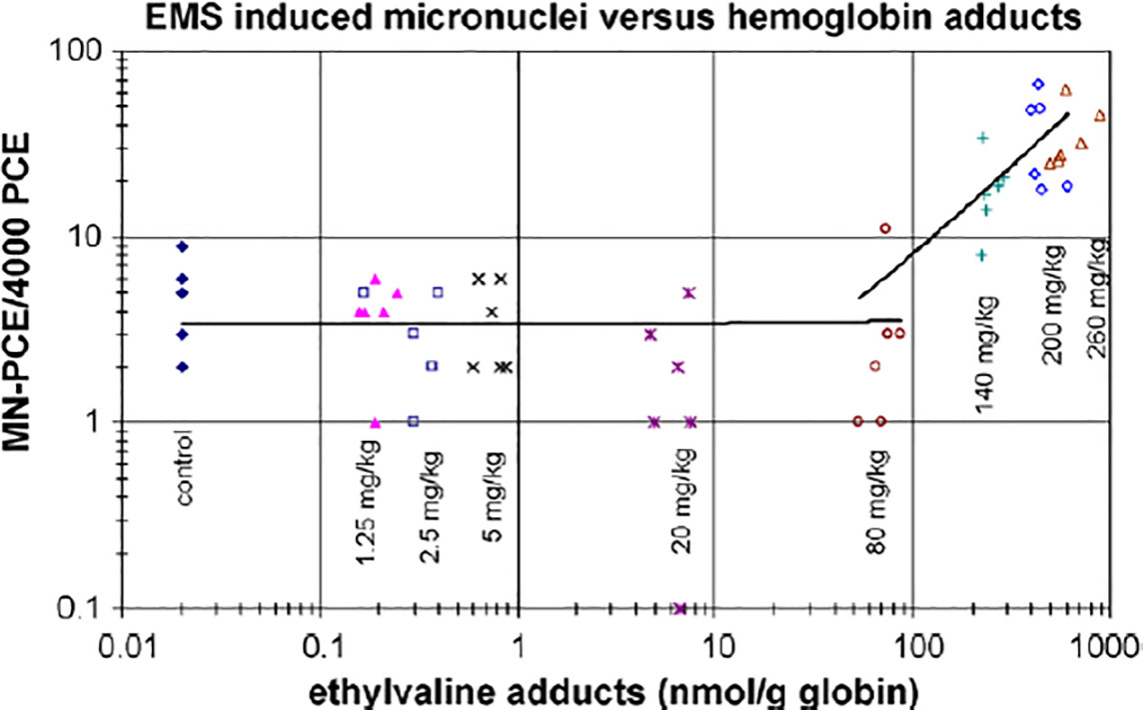
Reprinted with permission from Gocke and Müller, 2009, “Fig. 3. Frequencies of micronuclei as function of ethylvaline levels. MN‐PCE values are plotted for each mouse against the ethylvaline levels. Independent linear regression lines were fitted for animals receiving ≤80 mg/(kg day) and for animals receiving ≥80 mg/(kg day).”

Several recent *in vivo* datasets on nitrosamines and other direct‐acting mutagens focused on dose–response and contributed additional weight‐of‐evidence for non‐linear dose–response with DNA‐reactive chemicals. Perhaps the strongest dataset came from the Hoffmann‐LaRoche researchers, as it included internal dosimetry (hemoglobin adducts) in addition to gene mutation (Muta™Mouse) and MN induction as measures of *in vivo* genotoxic events, over a range of doses of EMS and ENU (see Fig. 9) (Gocke and Müller, 2009). The hydroxyethylvaline adducts clearly increased steadily with increasing EMS dose, as would the predicted *N*7‐EG adducts based on Murthy and colleagues (Murthy et al., 1984), representing an increasing internal dose. However, the markers of genotoxic effect did not increase above control levels until the EMS dose was above 20–50 and 80 mg/kg bw, for *lacZ* mutations and MN, respectively (Gocke and Müller, 2009). Several lower EMS doses did not result in any quantifiable increase in either gene mutation (*lacZ*) or MN levels. The data for ENU did not show the same threshold/BPD dose–response result, although the authors discuss how lower doses might have demonstrated a dose–response similar to EMS. Indeed later work did demonstrate such a threshold (and BPD) in rats for the *in vivo* induction of *pig‐A* mutations in erythrocytes following 28‐d repeated dosing with either EMS (21.9 mg/kg bw) or ENU (0.88 mg/kg bw), based on a better statistical fit for a threshold dose–response model (Dobo et al., 2011). Additional work with MNU and *pig‐A* demonstrated no increases of CD59^−^ reticulocytes or erythrocytes for repeated doses (28 d) of MNU (0.1 to 1.25 mg/kg), whereas the MN frequency was induced at doses above 0.3 mg/kg bw, again demonstrating a low‐dose region with no increases in genotoxic effects for *in vivo* tests (Lynch et al., 2011). Adding to the weight of evidence for non‐linear/threshold dose–responses, a similar dataset was obtained with 4‐nitroquinoline‐1‐oxide (4NQO), a direct‐acting genotoxicant, and *pig‐A*, where repeated exposures (28 d) to 4NQO at the lowest dose of 1.25 mg/kg bw also did not result in statistically significant increases in CD59^−^ reticulocytes or erythrocytes, nor in increased peripheral blood micronuclei (Stankowski et al., 2011). In fact, this kind of work was expanded beyond DNA‐reactive chemicals with the work of Olipitz and colleagues (Olipitz et al., 2012), who evaluated exposure of mice to low‐dose radiation and measured subsequent mutation induction, along with gene expression changes in several marker genes for DNA repair and cell cycle effects. Their data demonstrated that repeated whole body exposure to low doses of ionizing radiation (400‐fold background levels, 0.0002 cGy/min for 5 weeks) did not result in any significant increases in markers for genotoxic effects. They found no increases for the indirect radiation‐induced DNA adducts (8‐oxo‐dG, εdA, εdC), double strand breaks (DSBs), MN, or homologous recombination (HR), as assessed with the fluorescent yellow direct repeat reporter assay. They also did not find any increases in DNA damage response by gene expression of several markers for DNA repair. Historically, approaches to estimate cancer risk from chemicals have been based on methods derived from radiation data by the NAS (National Academy of Sciences, 1977), as described by Calabrese (Calabrese, 2013). Thus, identification of doses that do not result in significant increases in genotoxic effects for both DNA‐reactive chemicals and for radiation corroborates that there are low‐dose regions with no quantifiable increases in genotoxic effect responses, or “thresholds”, supporting determinations of PODs or BPDs.

The question of low‐dose linearity for mutation induction dose–response has also been investigated for the high production volume LMW chemical olefin/olefin oxide pairs, ethylene/ethylene oxide (E/EO) and propylene/propylene oxide (P/PO). These data add to the weight‐of‐evidence for “thresholds”/BPDs in the dose–response of adducts vs. mutations, especially the LMW *N*7‐alkylG adducts. Work conducted on E/EO and on P/PO provide insights on these questions with *in vivo* data, demonstrating that heavy burdens of *N*7‐alkylG adducts from repeated ethylene exposures (*N*7‐HEG) (Rusyn *et al*., 2005) or from repeated propylene (Pottenger et al., 2007) or PO exposures (*N*7‐HPG) (Rios‐Blanco et al., 2000) did not result in quantifiable increases in AP sites, a mechanism proposed to lead to mutation induction from depurination and inadequate repair of *N*7‐alkylG adducts (Neto et al., 1992). In addition, *in vivo* mutation assays of E‐ and P‐exposed rats (and mice for E) did not result in any increases in HPRT mutations (or MN), despite heavy *N*7‐alkylG adduct burdens (Walker et al., 2000, Pottenger et al., 2007). Data on *in vivo* mutation from EO itself show that exposures at or below 10–50 ppm EO do not result in any quantifiable increases in the genotoxic effect markers evaluated in somatic cells (HPRT mutations, reciprocal translocations, micronucleus), but increases were quantifiable at higher doses (Sisk et al., 1997, Walker et al., 1997, van Sittert et al., 2000, Recio et al., 2004, Donner et al., 2010). More recently an extensive assessment of *in vivo* mutation induction in mouse lung by repeated exposures to EO utilized the Allele‐specific Competitive Blocker Polymerase Chain Reaction approach to quantify dose–response for mutations induced in a cancer‐relevant gene, *Kras,* along with measuring adducts and transgenic mutations in mouse lung (Haber et al., 2013, Parsons et al., 2013, Manjanatha et al., 2017). The investigators reported that, despite dose‐dependent increases in *N*7‐HEG adducts, no statistically significant (*p* < 0.05) increases were found for *Kras* (or *cII*) mutations in mouse lung exposed to 10 ppm EO for 4 weeks of repeated exposure. There were statistically identified increases observed at 50 ppm in some tissues, although it is not clear whether these resulted from EO‐specific DNA adducts or from indirect mechanisms such as oxidative stress. Together these *in vivo* mutation studies consistently show non‐linear dose–response curves for induction of genotoxic/mutagenic effects, where low exposures result in quantifiably increased DNA adduct levels but no concomitant quantifiable increases in genotoxic/mutagenic effects.

### Integration with Contributions from International Workshops & Committees

Parallel to and following collection of the experimental data, it is necessary to interpret and integrate the new information. Analysis of approaches for applying these new data to risk assessment were topics of several international committees and workshops. The tripartite organization, ILSI/HESI, held a workshop in 2004 (Sander et al., 2005) as a kick‐off to an ILSI/HESI Project Committee on the Biological Significance of DNA Adducts. A framework for analysis and interpretation of DNA adduct data was developed (Himmelstein et al., 2009, Jarabek et al., 2009) and applied to three case studies (Tamoxifen, aflatoxin B1, and vinyl chloride) to demonstrate how DNA adduct data could be applied to MOA determination and risk assessment (Pottenger et al., 2014). Some key conclusions from this 10‐year effort included the following:

- “The ubiquitous existence of endogenous DNA adducts (often chemically identical with induced ones) must be considered in the overall assessment.
- Not all DNA adducts are considered pro‐mutagenic or lead to a heritable effect; an adduct is not equivalent to a mutation.
- Adduct data alone cannot be used to predict a quantitative cancer risk.
- Dosimetry considerations are critical; thus adduct data must be integrated with other relevant data, such as PK/TK, environmental and background exposures, adduct fate, system status (*e.g*., healthy or not), *etc*.
- While *in vitro* data can provide useful information, they cannot inform quantitative risk assessment if collected at concentrations that are unachievable for *in vivo* situations.
- To establish a DNA‐reactive MOA, it is necessary to demonstrate pro‐mutagenic DNA adducts in the target tissues for carcinogenicity; some mutations in critical genes (*e.g*., *p53; ras*) in tumors should be attributable to chemical‐specific DNA adducts detected in the target tissue.”

The work in the ILSI/HESI project committee dovetailed with the early work in a related ILSI/HESI Committee, the *In Vitro* Genetic Toxicity (IVGT), which later broadened its purview and became the Genetic Toxicology Technical Committee (GTTC), still working today. One key topic that this expert group addressed was quantitative dose–response modelling of genetic toxicology data, in an effort to bring genetic toxicology into the same realm as the rest of toxicology disciplines, developing quantitative methods to apply to dose–response of genotoxic effects to inform risk assessment. The ILSI/HESI GTTC carried out extensive dose–response analyses on all adequate *in vitro* and *in vivo* gene mutation and micronucleus dose–response datasets for MMS, EMS, MNU, and ENU that were published pre‐2012. In a report by Gollapudi and colleagues (Gollapudi et al., 2013), data available for EMS and MMS were assessed, and clear POD metrics were defined for each dose–response, with NOGEL, BPD/L, and BMD/L metrics being defined for each dose–response dataset. Available, adequate MNU and ENU dose–response data were then assessed in a second effort, using a wider range of statistical approaches (Johnson et al., 2014), in addition to those employed previously. This analysis showed that the bilinear modelling approach (Lutz and Lutz, 2009) had some major drawbacks (*e.g*., required extensive data), such that BPD/L metrics could only be defined in ~5% of the cases. However, when the BMD‐1SD and BMD‐10% approaches were used, BMD/L metrics were defined in all cases. These findings indicate that the ‘threshold’ dose–response modelling provided within the bilinear approach is less suitable for robustly defining a POD with a standard dataset. This along with other information, resulted in the ILSI/HESI GTTC and International Workshops on Genotoxicity Testing (IWGT) 2013 expert groups (MacGregor et al., 2015a, 2015b) recommending the BMD/L approach as best for defining POD metrics for genotoxic effects. One other major recommendation was that, although statistically defined ‘thresholds’ can be determined using large datasets with bilinear dose–response modelling, the BMD/L model allows for smaller numbers of animals to be used, and more precise POD metrics to be defined; however, this is done with acceptance that low levels of even DNA‐reactive genotoxicants pose negligible concern to the human population. Additional aspects of mutation dose–response were explored in a HESI‐sponsored workshop in 2014 (White and Johnson, 2016), including further analysis of metrics and comparing results across *in vivo* transgenic rodent models and tissues, with recommendations on how to use BMD/Ls to address potency from *in vivo* mutation data (Wills et al., 2017).

Other contributions to the data and discussions around these issues included several papers on related topics, such as a proposed description of a mutagenic MOA by Pottenger and Gollapudi that defined potential key events leading to genetic effects, and discussed determination of the MOA for no effects, at the NOGEL—why we do not see mutations or other genotoxic effects at doses where we have adducts (Pottenger and Gollapudi, 2010). One by Klapacz and colleagues (Klapacz et al., 2016) also provided insights on these issues stemming from a 2014 SOT symposium that raised similar questions about the mechanisms underpinning non‐linear/bilinear genotoxic dose‐responses. With a focus on networking of DNA repair pathways, they integrated information to illustrate the biological pathways and networks involved in repair and homeostatic processes for various types of DNA damage. Indeed, evaluation of these key cellular responses should be an integral part of research designed to provide MOAs for NOGELs and PODs for genotoxic effects for compounds of interest, including known mutagens.

## CONCLUDING REMARKS

Therefore, after 15+ years of work by a multitude of researchers on many fronts, and putting all this information together, it is time to ask: did we answer our three original questions:

- What does an increase in chemical‐specific DNA adducts indicate?
- With particular reference to low levels of DNA adducts, what data demonstrate a consequence or otherwise for human health?
- What status should DNA adduct measurements have in overall hazard and risk assessment?

Based on all the above, the answer is ‘yes, in large part’. Given the extensive amount of data now available on DNA adducts (both exogenous and endogenous), and on dose–response for the induction of genotoxic effects (*in vitro* and *in vivo*), coupled with the advancements in interpretation and methods for quantitative dose–response modelling and assessment allowing for the determination of PODs useful for risk assessment, and the progress in the mechanistic understanding of and determination of MOA, this large body of complementary work has provided the field with a different perspective on what is known and what else might be useful.

Overall, the collective, collaborative research efforts of the past 15+ years have raised the understanding of the biological mechanisms employed in DNA damage and repair and its consequences, resulting from new techniques and measurement capabilities. A wealth of data is available to explain why an adduct does not equal a mutation and to confirm the ubiquity of endogenously formed DNA adducts. These data help elucidate the MOAs of agents at their NOGEL values and in the regions of dose–response curves with no increase in genotoxic effects despite increased DNA adducts. They provide quantitative methods to derive BMD/Ls and other PODs for genotoxic effects, and examples of how such data can and cannot be used to inform risk assessment of chemicals that are DNA‐reactive. Indeed, future considerations should focus on the analysis using the BMD/L approach, as this is now recommended over the bilinear/threshold and NOGEL approaches (Gollapudi et al., 2013, Johnson et al., 2014). As data and additional examples continue to be developed and published, we reach a point where we have to ask how much information is enough to determine negligible concern (*e.g*., setting exposure limits), whether empirical PODs, such as a NOGEL, or a modelled POD, such as a BMD; perhaps the data now available on LMW *N*7‐alkylguanine adducts should lead the way. The 2008 ECETOC workshop (Pottenger et al., 2009b) was not able to reach consensus on the specific question of what level of DNA adducts would be considered as exposures associated with negligible risk. Certainly, with the increased current mechanistic understanding that was identified as necessary, coupled with the consensus that DNA adducts are considered biomarkers of exposure and not biomarkers of genotoxic effect, the conditions are ripe. Thus, with the additional site‐specific mutagenesis data demonstrating that *N*7‐HEG and *N*7‐HPG are not mutagenic, and all the additional *in vitro* and *in vivo* mutation datasets demonstrating clear NOGELs for direct‐acting mutagens (MNU, ENU, MMS, EMS, 4NQO, *etc*.), with more *in vivo* transgenic mutation data coming, we are ready to reach a consensus on a level of DNA adducts—perhaps it would only be for LMW *N*7‐alkylG adducts—that would be considered of negligible concern. This might even be applied in a similar way to the application of concentration cut‐off values with the Globally Harmonized System of classification approach, or possibly in potency determinations as discussed by Hennes et al. (2014). Questions such as how broadly these examples and this growing database can be extrapolated, and how many data are enough, are critical to address as a scientific body. With a goal of informing risk assessment, regulatory acceptance of these insights and application of them in regulatory situations remain a key challenge, one that can only be addressed by the regulatory community, itself, in applying the many learnings from the recent science as described above. The necessary tools, such as BMDs and PODs, and the data, are available and sufficiently mature for use by the regulatory community. We will all learn from that application.

**Abbreviations**
4NQO: 4‐nitroquinoline‐1‐oxide
8‐oxo‐dG: 8‐oxo‐7,8‐dihydro‐2′‐deoxyguanosine
AAG: 3‐alkyladenine DNA glycosylase
ACC: American Chemistry Council
alkyl‐FAPy: N^5^‐alkyl‐2,6‐diamino‐4‐hydroxy‐5‐formamidopyrimidine
alkylG: alkyl/hydroxyalkylguanine
AP site: abasic or an apurinic site
APNG: alkylpurine DNA *N*‐glycosylase
BER: base excision repair
BLEO: bleomycin
BMD: benchmark dose
BMD/L: benchmark dose lower bound
BPD: break point dose
BPDL: break point dose lower bound
Cefic: European Chemical Industry Association
DR: direct reversal
dsDNA: double‐stranded DNA
E: ethylene
ECETOC: European Centre for Ecotoxicology and Toxicology of Chemicals
EMS: ethyl methane sulfonate
ENU: ethyl nitrosourea
EO: Ethylene Oxide
FAPy: N^5^‐alkyl‐2,6,‐diamino‐4‐hydroxy‐5‐formamidopyrimidine
GTTC: Genetic Toxicology Technical Committee
HE: hydroxyethyl
HR: homologous recombination
ILSI/HESI: International Life Sciences Institute/Health and Environmental Sciences Institute
IVGT: In Vitro Genetic Toxicity Committee
JIG: Joint Industry Group
LMW: low molecular weight
MGMT: methylguanine methyl transferase
MMR: mismatch repair
MMS: methyl methane sulfonate
MN: micronucleus/micronuclei
MNU: methyl nitrosourea
MOA: mode of action
MPG: N‐methylpurine DNA glycosylase
*N*1‐HEdA: *N*1‐(2‐hydroxyethyl)adenine
*N*3‐HEA: *N*3‐(2‐hydroxyethyl)adenine
*N*7‐alkylG: N7‐alkyl/hydroxyalkylguanine
*N*7‐HPG: N7‐(2‐hydroxypropyl)guanine
*N*7‐MG: N7‐methylguanine
*N*7‐OEG: *N*7‐(2‐oxoethyl)guanine
NAS: National Academy of Sciences
NER: nucleotide excision repair
NOEL: no observed effect level
NOGEL: no observed genotoxic effect level
O^4^‐ET: O^4^‐ethylthymidine
O^6^‐HEG: O^6^‐hydroxyethylguanine
O^6^‐1HPdG: O^6^‐(2‐hydroxypropyl)‐2′‐deoxyguanosine
O^6^‐2HPdG: O^6^‐(2‐hydroxy‐(1‐methyl)‐ethyl)‐2′‐deoxyguanosine
O^6^‐HPG: O^6^‐hydroxypropylguanine
O^6^‐MG: O^6^‐methylguanine
P: propylene
PO: propylene oxide
POD: point of departure
ROS: reactive oxygen species
TLS: translesion synthesis

## Footnotes

1 The JIG was comprised of the ACC Olefins Panel Ethylene/Propylene Work Group (E/PWG); the ACC Propylene Oxide/Propylene Glycol (PO/PG) Panel; the ACC Ethylene Oxide Industry Council (EOIC); the Cefic Lower Olefins Sector Group (LOSG); the Cefic PO & Derivatives SG (PO & Der SG); and the Cefic EO & Der SG. Participating groups determined which projects were of interest to them and co‐funded accordingly.

2 The 2001 workshop was funded by the Cefic LOSG and the Global PO Research Group (ACC PO/PG Panel and Cefic PO & Der SG).

3 Some examples of *in vivo* datasets that demonstrate thresholds for genotoxic mutagens/carcinogens include the work on EMS published by (Gocke and Müller, 2009), and *in vivo* data on MMS published by (Ji *et al*., 2016), which demonstrates a threshold for induction of micronucleus.
